# Extended phenotype in evolutionary medicine

**DOI:** 10.1093/emph/eoz009

**Published:** 2019-03-12

**Authors:** Stephen I Valentino, Neil S Greenspan

**Affiliations:** 1 Department of Nutrition, Case Western Reserve University, Cleveland, OH, USA; 2 Department of Pathology, Case Western Reserve University, Cleveland, OH, USA

## DEFINITION AND BACKGROUND

**Figure 1.**
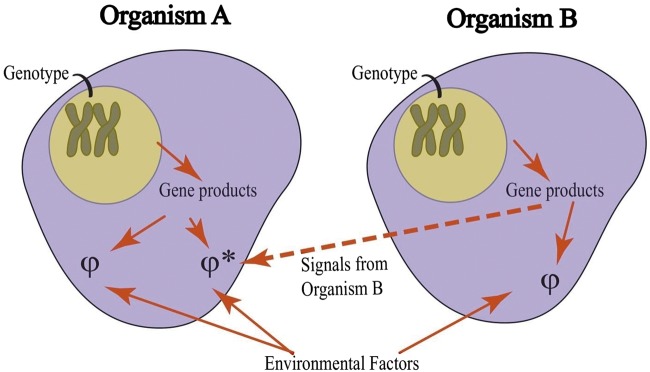
Extended phenotype schematized. The standard genotype–phenotype (Φ) relationship is illustrated for Organism A without (left, Φ) and with (right, Φ*) influence mediated, directly or indirectly, via molecules encoded by genes in Organism B (far right). So, one or more genes of Organism B modify the phenotype of Organism A, i.e. genes in B are causally related to extended phenotypic effects manifested by A

The human phenotype is conventionally conceived of as resulting from the transcription and, for protein-encoding genes, translation of the sequences inherited from parents in conjunction with environmental factors. In 1982, Dawkins [1] defined the ‘extended phenotype’ attributable to a given gene as effects associated with the corresponding DNA sequence that are manifested by an organism whose cells do not contain that gene, i.e. the phenotype of Organism A can be influenced by genes in the cells of Organism B (Fig. 1) and sometimes in ways that enhance the fitness of B.

## EXAMPLES IN HUMAN BIOLOGY AND PUBLIC HEALTH

Extended phenotypic effects can involve organisms of the same or different species. For example, the *Lactobacillus acidophilus* genome encodes gene products that produce a metabolite able to mediate signals between cells of this symbiotic species that enable better adherence to gut eipthelium [2]. An example of a *trans*-specific ‘partnership’ is provided by *Bacteroides thetaiotaomicron*, which carries genes encoding proteins able to synthesize metabolites that guide host gut development [3].

## EXAMPLES IN CLINICAL MEDICINE

Perhaps the most thoroughly explored examples of extended phenotypes relevant to medicine derive from the genes of pathogens that manipulate host phenotypes. An example is provided by the *nef* gene, which encodes the Nef protein of HIV-1. The Nef protein decreases the number Human Leukocyte Antigen (HLA) class I molecules on the plasma membranes of CD4^+^ T cells [4]. By reducing the availability of HLA class I molecules presenting peptides derived from HIV antigens, the virus likely decreases the ability of CD8^+^ T cells to cause the lysis of infected CD4^+^ T cells [5]. This form of immune escape would engender greater replication of the virus and therefore is likely to be subject to strong selection.

Based on already published studies, there are likely to be numerous instances in which genes in fellow humans or other organisms, such as microbial species, influence the risk for or manifestations of one or another disorder in a particular human individual. One can readily imagine complex networks of such health influencing effects. Therefore, the phenomenon of extended phenotype is likely to be highly relevant to medicine.

## FUNDING

Neil S. Greenspan is supported by the CWRU/UH Center for AIDS Research: NIH Center for AIDS Research grant P30 AI036219.
